# Study of inelastic nuclear interactions of 400 GeV/c protons in bent silicon crystals for beam steering purposes

**DOI:** 10.1140/epjc/s10052-018-5985-8

**Published:** 2018-06-18

**Authors:** W. Scandale, F. Andrisani, G. Arduini, F. Cerutti, M. Garattini, S. Gilardoni, A. Masi, D. Mirarchi, S. Montesano, S. Petrucci, S. Redaelli, P. Schoofs, R. Rossi, D. Breton, D. Chaumat, S. Dubos, J. Maalmi, A. Natochii, V. Puill, A. Stocchi, E. Bagli, L. Bandiera, G. Germogli, V. Guidi, A. Mazzolari, F. Murtas, F. Addesa, G. Cavoto, F. Iacoangeli, F. Galluccio, A. G. Afonin, Yu. A. Chesnokov, A. A. Durum, V. A. Maisheev, Yu. E. Sandomirskiy, A. A. Yanovich, A. D. Kovalenko, A. M. Taratin, G. I. Smirnov, A. S. Desinov, Yu. A. Gavrikov, Yu. M. Ivanov, L. P. Lapina, L. G. Malyarenko, V. V. Skorobogatov, J. Fulcher, T. James, G. Hall, M. Pesaresi, M. Raymond

**Affiliations:** 1 CERN, European Organization for Nuclear Research, 1211 Geneva 23, Switzerland; 2 Laboratoire de l’Accélérateur Linéaire (LAL), Université Paris Sud Orsay, Orsay, France; 3 Dipartimento di Fisica e Scienze della Terra, INFN Sezione di Ferrara, Universita di Ferrara, Via Sarogat 1 Blocco C, 44121 Ferrara, Italy; 4 INFN, Laboratori Nazionali di Frascati, Via Fermi, 40, 00044 Frascati, Rome Italy; 5 INFN Sezione di Roma, Piazzale Aldo Moro 2, 00185 Rome, Italy; 6 Dipartimento di Fisica, Sapienza Univ. Roma, Piazzale A.Moro, 2, 00185 Rome, Italy; 7 INFN Sezione di Napoli, Complesso Universitario di Monte Sant’Angelo, Via Cintia, 80126 Naples, Italy; 8 NRC Kurchatov Institute-IHEP, 142281 Protvino, Russia; 9 Joint Institute for Nuclear Research, Joliot-Curie 6, 141980 Dubna, Russia; 10 Petersburg Nuclear Physics Institute in National Research Centre “Kurchatov Institute”, 188300 Gatchina, Russia; 11 Imperial College, London, UK; 12 Taras Shevchenko National University of Kyiv (TSNUK), Kiev, Ukraine

## Abstract

Inelastic nuclear interaction probability of 400 GeV/c protons interacting with bent silicon crystals was investigated, in particular for both types of crystals installed at the CERN Large Hadron Collider for beam collimation purposes. In comparison to amorphous scattering interaction, in planar channeling this probability is $\sim 36\%$ for the quasi-mosaic type (planes (111)), and $\sim 27\%$ for the strip type (planes (110)). Moreover, the absolute inelastic nuclear interaction probability in the axial channeling orientation, along the $\langle 110\rangle$ axis, was estimated for the first time, finding a value of $0.6\%$ for a crystal 2 mm long along the beam direction, with a bending angle of 55 $\upmu$rad. This value is more than two times lower with respect to the planar channeling orientation of the same crystal, and increases with the vertical angular misalignment. Finally, the correlation between the inelastic nuclear interaction probability in the planar channeling and the silicon crystal curvature is reported.

## Introduction

**Table 1:** Crystal parameters: name, physical dimensions ($L_{\mathrm{X}}$, $L_{\mathrm{Y}}$, $L_{\mathrm{Z}}$), bending angle ($\theta$), PCH channeling efficiency and type of the crystal

Crystal	$L_{\mathrm{X}}$ (mm)	$L_{\mathrm{Y}}$ (mm)	$L_{\mathrm{Z}}$ (mm)	$\theta$ ($\upmu$rad)	Eff. ($\%$)	Type
	(±0.02)	(±0.1)	(±0.02)	(±2)	(±1)	
QMP46	30.00	27.0	4.00	50	66	LHC 2nd gen.
STF76	0.50	55.0	4.00	65	74	LHC 1st gen.
STF99	2.01	55.0	2.01	120	64	INI (PCH)
STF100	1.02	55.0	1.95	63	76	INI (PCH)
STF101	1.01	55.0	1.99	165	58	INI (PCH)
STF103	1.97	55.0	1.88	55	75	INI (ACH)
STF105	0.55	55.0	4.07	50	74	LHC 2nd gen.

In the last decade, the UA9 Collaboration investigated a new approach to collimation in hadron accelerating machines, using the planar channeling (PCH) process realized in bent silicon crystals [1–6]. High-energy charged particles impinging on the crystal with small angles relative to the lattice planes move oscillating between two neighboring planes, and consequently can be deflected by the bend angle. In this condition, close collisions with the crystal atoms are strongly suppressed [7]. Following this principle, a bent crystal can be used as a primary collimator for the Large Hadron Collider (LHC) at CERN, deflecting the beam halo particles directly onto an absorber.

Since 2015, UA9 has obtained very encouraging results in the LHC, performing channeling at the record energy of 6.5 TeV [8] and observing a strong reduction of beam losses due to Inelastic Nuclear Interactions (INI) in the aligned crystal in comparison with its amorphous (AM) orientation. AM orientation, typically far away from PCH orientation, is the condition in which the bent mono-crystalline silicon can be considered disordered as amorphous silicon. For this purpose, the study of INI produced by high energy particles in the LHC crystals has an influential role for the estimation of the beam losses in the machine. In particular, it is important to estimate the reduction factor in PCH orientation, used for crystal collimation, with respect to the AM orientation.

A first general study on the INI topic was published by the UA9 Collaboration in 2010 [9]; however further and more detailed studies were required. For this purpose, in 2015, a large number of INI events produced by 400 GeV/c protons have been collected at the H8 SPS extraction line, using an upgraded experimental apparatus (Sect. 2). Different types of silicon crystal were tested, including crystals for the LHC, crystals with different curvatures and others optimised for axial channeling (ACH) studies [10, 11]. The results of the first analysis, which shows the INI rate of channeling along the $\langle 110\rangle$ with respect to the $\langle 111\rangle$ axis case, are presented in [12].

Recently, the UA9 Collaboration performed a further independent analysis of these data, using a more sophisticated approach, able to estimate the absolute INI probability, and with the purpose to look deeper into the results (Sect. 3). This latest study is presented in this paper. In particular, the results for two different types of crystal technology installed in LHC, quasi-mosaic and strip crystals, are reported. A comparison between their first generation, installed in LHC ring 1 in 2014, with the second one, studied for the installation in the LHC ring 2 in 2016, was performed.

Moreover, the new approach applied to the data presented in [12] has allowed an estimate of the absolute INI probability in ACH for the first time. The behaviour of the INI rate varying the vertical angular orientation of the crystal with respect to the best axial orientation is also shown (Sect. 5).

Finally, an estimation of the INI reduction factor in PCH as a function of the crystal curvature, is presented (Sect. 6).

These studies are helpful for better understanding of bent-crystal physics, not only for collimation purposes, but also for other kinds of beam manipulation, such as extraction [13], splitting, focusing, and defocusing [14].

## Experimental measurements

The standard UA9 procedure to test bent crystals at the H8 SPS beam extraction line implies the use of a dedicated tracker to reconstruct individually the tracks of particles before and after the interaction with the crystal. In this way, it is possible to measure the deflection angle and the efficiency of the channeling process. The tracker is composed of 5 planes, 2 upstream and 3 downstream of the crystal position, and it reconstructs the tracks in an arm length of 10 m for both incoming and outgoing particles. Each plane is composed of 2 microstrip silicon sensors. For 400 GeV/c protons, the system has an excellent angular resolution of 2.8 $\upmu$rad for both incoming and outgoing tracks, and a cumulative angular resolution of 5.2 $\upmu$rad for a single deflection event. This is mainly due to multiple scattering in the sensor layers [15].

Results concerning one quasi-mosaic silicon crystal, produced and bent as described in [16], and six strip silicon crystals produced and bent with the method described in [17–20] are reported here.

The main crystal parameters are listed in Table 1. Every measurement has been taken with a 400 GeV/c proton micro-beam, with the following parameters: $\sigma _x\simeq 1\hbox { mm}$, $\sigma _{y}\simeq 1\hbox { mm}$, $\sigma _{\theta _{x}}\simeq 10\,\upmu \hbox {rad}$, $\sigma _{\theta _{y}}\simeq 10\,\upmu \hbox {rad}$.

**Fig. 1:**
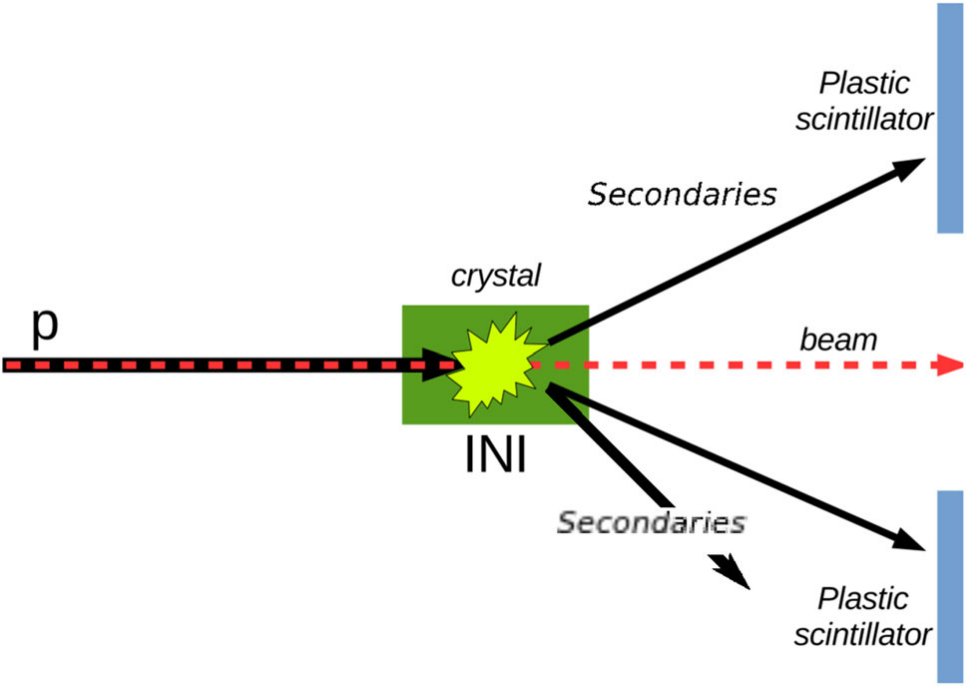
Scheme of the INI measurement principle

The basic idea of these measurements is to use two fast plastic scintillators [21], integrated in the tracker DAQ system, to count the secondary particles produced by INI, linking each of these events in coincidence with the corresponding tracker event. In this way, it is possible to analyse only the events that have a single incoming track, and multiple outgoing tracks after an interaction in the crystal, excluding a priori several spurious events. The scintillators are positioned on both sides of the beam line, symmetrically with respect to the beam axis (Fig. 1).

The distance between the crystal and the scintillators along the beam direction is 24.5 cm and it is fixed for all measurements reported here. A transverse gap between the two scintillators, of the order of 1 cm, is left to avoid any signals from beam particles. This gap varies slightly for the different measurements, as explained in the next section. The small dimensions of the two scintillators ($10 \times 20 \times 4 \hbox {mm}^3$) and a very short coincidence time gate between them ($\sim$ 3 ns) allow to suppress a large part of the background, that results mainly from interactions of the beam with upstream collimators and other components of the H8 line. To estimate the residual background, an alignment run without the crystal was performed before each high statistics data-taking run, and the background count is subtracted in the data analysis. This last background estimation is necessary to remove the INI signal of the beam particles with the second tracker plane and the air upstream of the crystal, including the backscattering component, that cannot be excluded by selecting only single incident particle tracks. A check was made using the signal coming from the particles that did not impinge on the crystal during high statistics data-taking runs, obtaining extremely compatible results. This is a further confirmation of the good quality of the data acquired and analysed.

The INI rates ($f_{\mathrm{INI}}$) are then computed, dividing the number of INI events ($N_{\mathrm{INI}}$) detected by the number of incoming tracks impinging on the crystal ($N_{\mathrm{in}}$), for each specific orientation:

1 $$\begin{aligned} f_{\mathrm{INI}}=\frac{N_{\mathrm{INI}}}{N_{\mathrm{in}}}. \end{aligned}$$

## Simulations

**Fig. 2:**
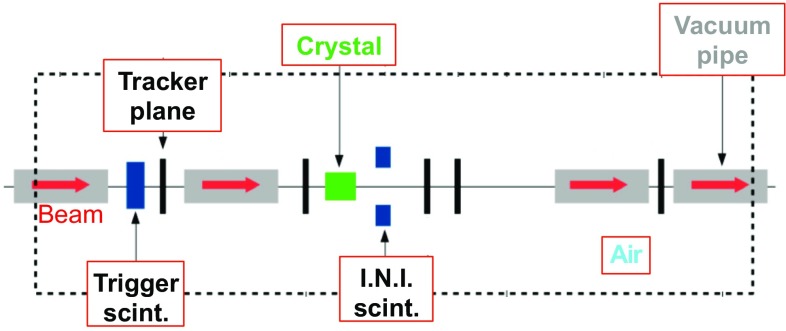
Schematic view of the H8 layout simulated to estimate the $E_{\mathrm{INI}}$ factor

In the previous section, the technique used to measure the INI rate was described, but another step is necessary to estimate the related probability. The experimental setup is not able to register all the INI events generated in the crystal lattice, so its geometry and the detection efficiency affect the measurements and have to be considered during the analysis. To do that, the experimental setup (Fig. 2) has been simulated, computing INI events occurring in the crystal ($S_{\mathrm{INI}}$) and the corresponding secondary events detected by the apparatus ($S_{\mathrm{det}}$). The ratio between these two numbers is our detection efficiency factor:

2 $$\begin{aligned} E_{\mathrm{INI}}=\frac{S_{\mathrm{det}}}{S_{\mathrm{INI}}}. \end{aligned}$$

$S_{\mathrm{det}}$ includes also the detection efficiency of the scintillators, considering as representative events only those that produced an energy deposition above the minimum ionising particle threshold. Above this threshold, the scintillators can be considered very close to 100$\%$ efficient.

For each crystal measurement, the factor $E_{\mathrm{INI}}$ was estimated with FLUKA [22, 23], using a customized user routine, and also with GEANT4 [24–26], with an independent blind approach. The compatibility is of the order of 1$\%$. For different measurements, $E_{\mathrm{INI}}$ can vary because it depends on the kind of crystal-holder system, on the gap between scintillators, and on the beam axis position with respect to the centre of the gap itself. The related error is $\sim 1\%$. Several tests with the same crystal, using different gaps and different beam axis positions, were successfully performed to check the reliability of this simulation tool. Computing and applying appropriate efficiency factors for different configurations, the INI probabilities are compatible within the error bars.

The INI probability is then simply obtained subtracting the background from the measured rates, and dividing by the $E_{\mathrm{INI}}$ factor.

3 $$\begin{aligned} P_{\mathrm{INI}}=\frac{f_{\mathrm{INI}} - f_{\mathrm{BKG}}}{E_{\mathrm{INI}}}. \end{aligned}$$

This simulation tool has also been very useful to study the most efficient configuration of the experimental apparatus, computing the optimal sizes, positions and thresholds of the two scintillators, allowing to maximise the signal-to-background ratio. The same approach was followed to design a new apparatus for future measurements with positive pions and different kinds of heavy ions in the crystal, at different energies.

## INI probabilities in LHC crystals

With the objective of studying the beam losses produced by the crystal in the LHC, INI rate measurements on both types of the second generation of LHC crystals (QMP46 and STF105) were performed. They were two of the best candidates for the installation in LHC ring 2 in 2016.

In the following plots, rates and probabilities are shown as a function of angular cut ($\pm \theta$) around the crystal PCH or ACH best orientation, from which incident particles are included in the analysis. For 400 GeV/c protons, the channeling acceptance angle, called the critical angle $\theta _C$, is $\sim$10 $\upmu$rad. For this reason, to have a conservative approach, the angular cut taken as a reference for all the results reported in this paper, is $\theta _C/2 = 5\,\upmu$rad [7].

**Fig. 3:**
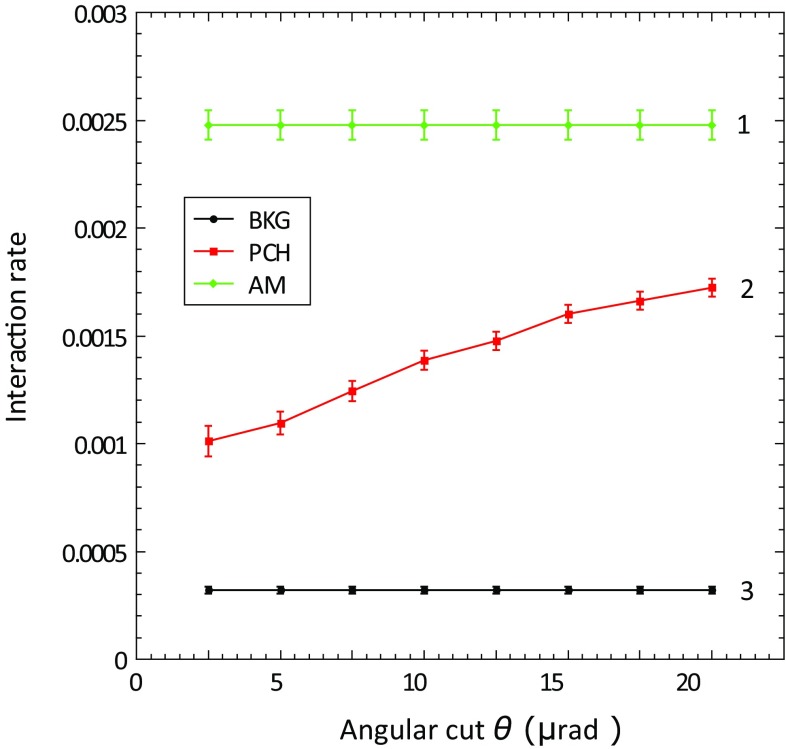
The INI rate measured for the QMP46 crystal as a function of the horizontal angular cut $\theta$ applied in the selection of incident 400 GeV/c protons for the amorphous orientation (1), the planar channeling orientation (2), and the case without the crystal in the beam (experimental background) (3)

**Fig. 4:**
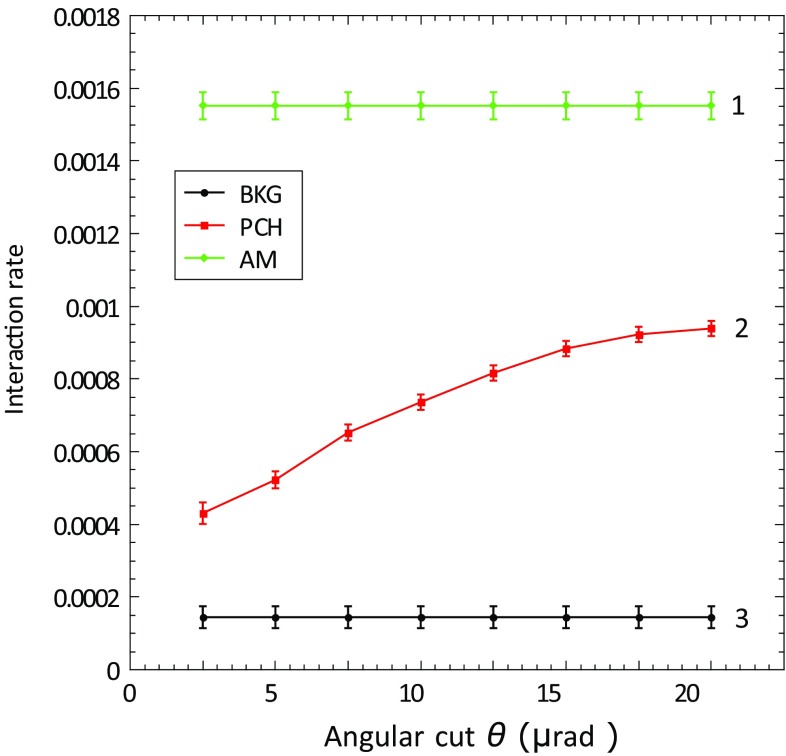
The INI rate measured for the STF105 crystal as a function of the horizontal angular cut $\theta$ applied in the selection of incident 400 GeV/c protons for the amorphous orientation (1), the planar channeling orientation (2), and the case without the crystal in the beam (experimental background) (3)

In Figs. 3 and 4, INI rate plots are displayed, presenting the comparison between AM and PCH orientations and the background, for QMP46 and STF105 respectively.

**Fig. 5:**
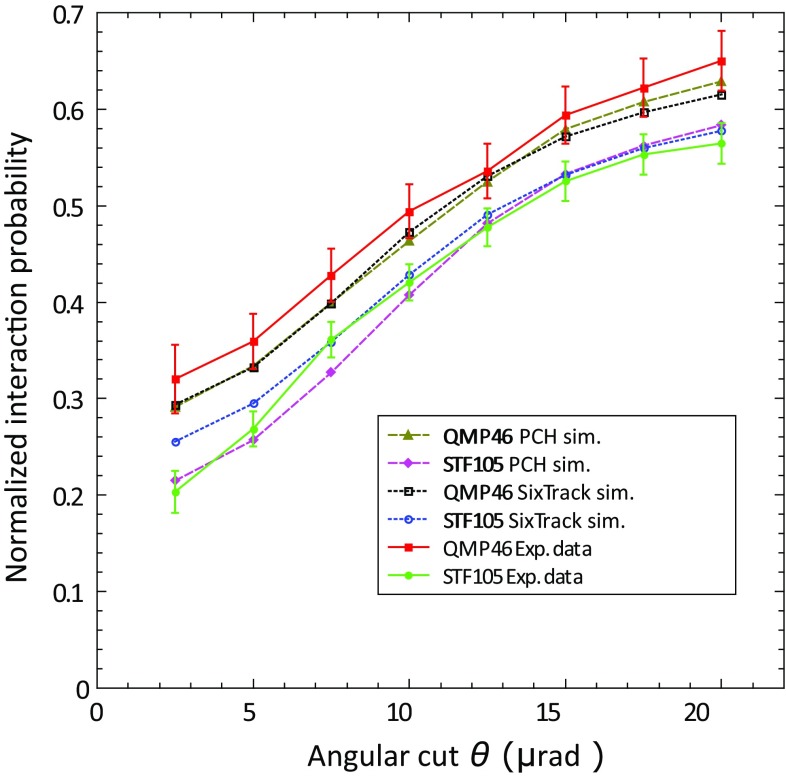
Comparison of the INI probability in PCH (normalised to the related AM value) between the QMP46 and STF105 crystals, as a function of the angular cut $\theta$ applied in the selection of incident 400 GeV/c protons. The simulation results of the SixTrack crystal routine and the Planar Channeling simulations are shown

In addition, Fig. 5 is showing the comparison of the INI probability in the two crystals. The probabilities are normalised to the respective AM value.

From the plots, it is evident that the quasi-mosaic crystal shows a higher value of INI normalised probability in channeling orientation ($\sim 36\%$), in comparison to the strip crystal ($\sim 27\%$). Considering that the two crystals have the same bending angle ($\sim 50$ $\upmu$rad) and the same thickness (4 mm), this difference can be explained only by the different crystalline plane orientation used to achieve channeling, (111) for the quasi-mosaic, and (110) for the strip. The average distance between (111) planes (1.568 Å) is smaller than for (110) planes (1.92 Å), and consequently the probability that a particle has an INI with the lattice atoms is higher [12].

An important parameter to estimate the behaviour of a bent crystal is the reduction factor of the INI rate in AM and PCH orientations, which is defined as:

4 $$\begin{aligned} R_{\mathrm{AM/(PCH)}}=\frac{P_{\mathrm{INI}}\mathrm {(AM)}}{P_{\mathrm{INI}}\mathrm {(PCH)}}. \end{aligned}$$

In this case, the reduction factor, for a 5 $\upmu$rad angular cut, is $3.7\pm 0.1$ for the strip crystal, and $2.8\pm 0.1$ for the quasi-mosaic one. This difference is very well confirmed by the Planar Channeling full analytical routine [27] simulations, shown in the plot, and compatible with the data within the error bars. Also the SixTrack Crystal routine [6], benchmarked on experimental data and optimised to be compatible with detailed multi-turn simulations in large circular accelerators, provides good description of the data.

In addition, the comparison between the STF76 (first LHC generation) and the STF105 (second LHC generation) was performed. The new generation of two types of LHC crystals has a better resistance to the LHC pre-installation heating process, and, in particular, the STF105 has a new better performing bending holder. The STF76 has the same thickness (4 mm), but a larger deflection angle ($\sim 65$ $\upmu$ rad), with respect to the STF105 ($\sim 50$ $\upmu$rad). This small difference appears as a slightly higher value for INI rate in the channeling orientation, as expected, but anyway within the error bars. From the point of view of INI probability, it is possible to conclude that the new holder system insignificantly changes the INI rate, and the reduction factor within 5 $\upmu$rad is compatible with the first generation one.

## Estimation of INI absolute probability for the axial channeling process

**Fig. 6:**
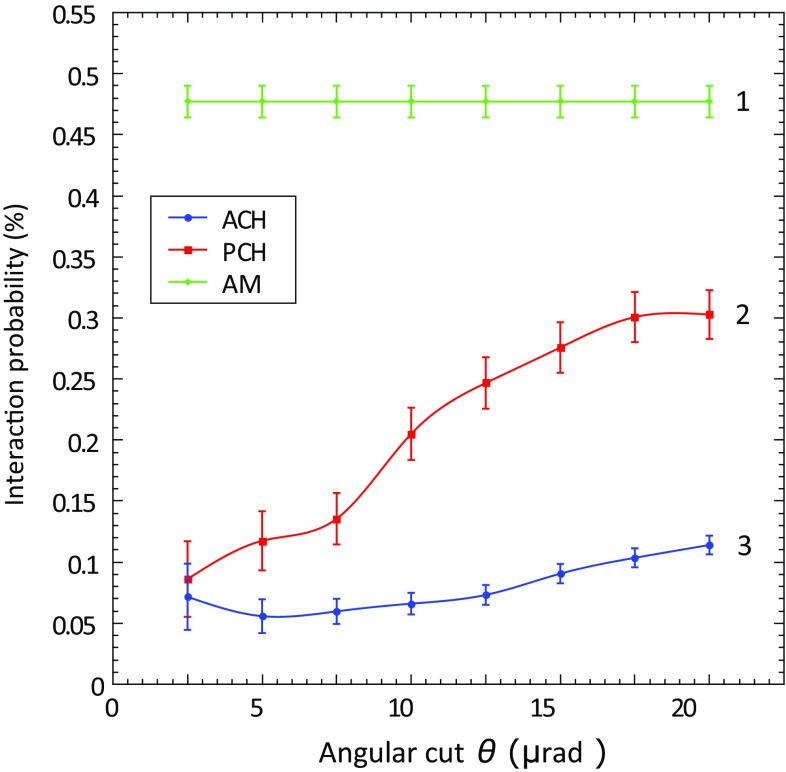
INI absolute probability for the STF103 crystal as a function of the circular angular cut $\theta$ applied in the selection of incident 400 GeV/c protons for the AM orientation (1), the PCH orientation (2), and the ACH orientation (3)

When a charged particle entering the crystal is well aligned with one of the crystallographic axis, it is subject to the ACH process, mainly characterised by the randomization of transverse momenta of the particles because of multiple scattering by atomic strings [10, 11]. It is obtained by aligning the crystal not only in the horizontal plane but also in the vertical one, along the main crystal axis. In this specific case, the crystal studied is the STF103 (see Table 1) with $\langle 110\rangle$ as the main axis. As already shown in [12], the INI rate in the ACH orientation is lower with respect to the PCH orientation. In Fig. 6, an estimate of the absolute INI probability is also shown, using the approach described in Sect. 4. For the ACH plot, the angular cuts ($\pm \theta$) are computed in cylindrical coordinates, taking into account that the ACH has a two dimensional acceptance:

5 $$\begin{aligned} \theta =\sqrt{\theta _{x_0}^2 + \theta _{y_0}^2}. \end{aligned}$$

In this case, the $E_{\mathrm{INI}}$ is 0.19 for a gap of 8 mm between the INI scintillators. Within 5 $\upmu$rad, the reduction factor AM/PCH is $4.3\pm 0.1$, while the value for AM/ACH is $8.3\pm 0.1$, almost double with respect to the planar case. This behaviour was predicted in [28].

Moreover, with an angular acceptance four times larger than the critical angle, it is interesting to note that the INI rate is more uniform in the ACH case, with respect to the PCH case. This larger acceptance of the axial process could be explained by the stronger electric fields of the crystal axes and consequently the larger critical channeling angle.

The SixTrack Crystal routine simulation for an angular cut of 5 $\upmu$rad gives an absolute probability of $\sim$ 0.1, matching the data for the PCH case. Note also the consistency with the results already obtained and published in 2010, studying a crystal of the same thickness (2 mm), but with a bending angle of $\sim$ 189 $\upmu$rad [9].

Despite better performance, in terms of INI reduction factor and acceptance with respect to the PCH one, unfortunately, the ACH process is not yet usable for the LHC collimation, both for technological and operational reasons. It needs a second degree of freedom in the alignment of the crystal with respect to the beam envelope, that means the use of a two coupled axis goniometer with a 0.1 $\upmu$rad angular resolution. Moreover, it would be necessary to find, monitor and preserve the ACH orientation of the crystal in a circular machine with dynamic cycle phases.

**Fig. 7:**
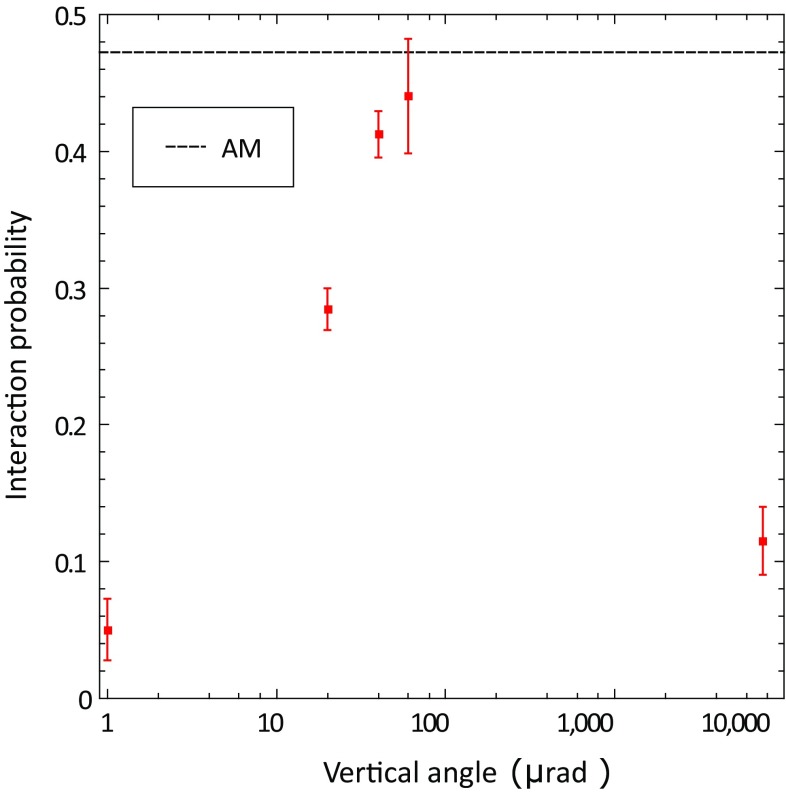
INI absolute probability for the STF103 crystal as a function of the vertical angular orientation with respect to the beam axis direction, keeping the horizontal one fixed in PCH. The first and the last points are related to the ACH and PCH orientation, respectively. The dotted line indicates the AM level

Another interesting study which has been performed with the STF103 crystal, is the variation of the INI probability in the crystal with the vertical angular orientation with respect to the beam axis direction, fixing the horizontal one in PCH. In Fig. 7, the INI absolute probabilities as a function of the vertical angle are displayed. The dotted line indicates the AM value (the same as in Fig. 6). In the transition region between ACH and PCH, unstable planar channeling is realized, and INI probability increases with vertical angle almost reaching the AM level around 60 $\upmu$rad, and then decreases again to the value of the PCH at 7.5 mrad from ACH. This features of the data reproduce qualitatively the theoretical and simulation studies performed in similar conditions and reported in [28]. Further measurements are planned in the next future to cover the region in which the INI probability is expected to decrease, trying to obtain more quantitative agreement in the full angular range.

## INI channeling reduction factor as a function of crystal curvature

**Fig. 8:**
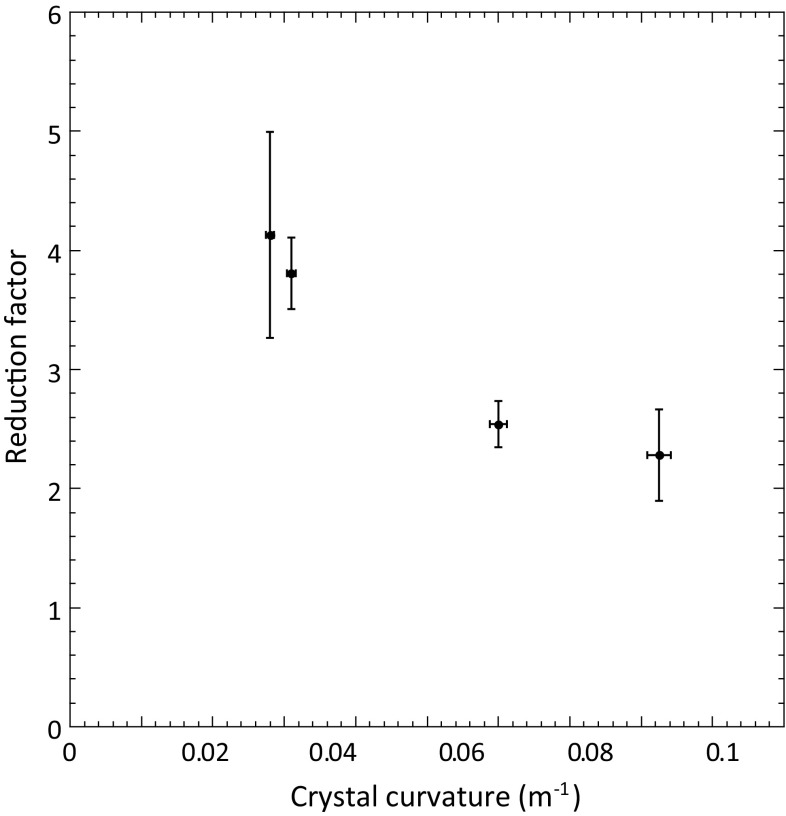
Dependence of the INI reduction factor (AM/PCH) as a function of the crystal curvature. For all the crystals reported, the thickness along the beam direction is 2 mm. The reduction factor has been computed with a horizontal angular cut value of incident 400 GeV/c protons equal to ± 5 $\upmu$rad

A specific study concerning the behaviour of the INI reduction factor (AM/PCH), varying the silicon crystal curvature, was performed. Four crystals, STF99, STFF100, STF101, STFF103 (Table 1), with the same thickness along the beam direction (2 mm), but with a different curvature, were measured. From the results shown in Fig. 8, it is evident that the reduction factor AM/PCH (Y axis) decreases if the crystal curvature increases. This means, that the same beam produces a smaller number of INI events in a crystal with smaller curvature, because the channeling fraction increases, as expected from the theory and simulations [7].

## Conclusions

Summarising, an extensive study of INI rate of 400 GeV/c protons with different bent silicon crystals has been performed. Thanks to a very well performing experimental apparatus and its FLUKA simulation, not only the INI rate, but also the absolute probability, have been estimated. Experimental results were successfully reproduced by the simulation implemented in SixTrack, which is used for beam dynamics simulations of the collimation process in the LHC. This gives us high confidence in the quality of theoretical studies focused on possible improvements of the LHC proton collimation assisted by bent crystals. Also, the comparison with the Planar Channeling full analytical simulation tool shows very good compatibility. In particular, the technologies for the quasi-mosaic and strip deflector production developed for the installation in LHC ring 2 have been compared. Both technologies show a strong reduction of INI in PCH orientation, with respect to the AM orientation. For the strip crystal, the reduction factor measured is $3.7\pm 0.1$, for the quasi-mosaic one it is $2.8\pm 0.1$. Further, a comparison between the first and the second generation of LHC crystals does not show any significant differences, confirming the same reduction factor.

Moreover, for the first time the estimation of the INI absolute probability for a silicon crystal optimised for ACH along the $\langle 110\rangle$ axis is shown. It has been obtained that a reduction factor in ACH is about twice higher than in PCH, confirming the results obtained in terms of rate in [12] and in good agreement with the results published in 2010 [9]. Another interesting result is the experimental evidence that INI probability varies with the vertical misalignment from the ACH orientation, with a peak around 60 $\upmu$rad from ACH, also if the crystal is horizontally oriented in PCH, reproducing qualitatively the theoretical and simulated results published in [28]. An additional new result is the measurement of the AM/PCH reduction factor as a function of the crystal curvature. As expected from the theory and simulations, the reduction decreases as the curvature increases [7].

Concluding, an efficient experimental setup to measure the INI frequencies in bent silicon crystals, and a reliable analysis tool to estimate the related INI probability, have been implemented. These studies are essential for a better understanding of the behaviour of bent silicon crystals used as LHC collimators, but also to investigate their physical features for different future applications in the beam steering context. The next step will be the study of INI probability of pions and heavy ions in bent crystals, at different energies.
